# Editorial: Multicomponent reactions (MCRs) towards scaffolds with versatile applications

**DOI:** 10.3389/fchem.2023.1332672

**Published:** 2023-11-20

**Authors:** Latifa Bouissane, Javier Echeverria, Toshifumi Dohi, Thomas J. J. Müller

**Affiliations:** ^1^ Molecular Chemistry, Materials and Catalysis Laboratory, Faculty of Sciences and Technologies, Sultan Moulay Slimane University, Beni-Mellal, Morocco; ^2^ Departamento de Ciencias del Ambiente, Facultad de Química y Biología, Universidad de Santiago de Chile, Santiago, Chile; ^3^ Graduate School of Pharmaceutical Sciences, Ritsumeikan University, Kusatsu, Shiga, Japan; ^4^ Institute of Organic Chemistry and Macromolecular Chemistry, Heinrich Heine University Düsseldorf, Düsseldorf, Germany

**Keywords:** multicomponent reactions (MCRs), one-pot reactions, diversity-oriented synthesis (DOS), consecutive processes, sequential processes, domino reactions

For a long time, multicomponent reactions (MCRs) have been known in the quickly developing field of organic chemistry. Already in 1850 Strecker reported the first three-component synthesis of α-amino nitriles (Strecker, 1850) which later on became a powerful entry to the steadily increasing demand for synthetic α-amino acids (Shibasaki et al., 2008; Wang et al., 2011), key building blocks for peptides, pharmaceuticals, and food additives. However, for a long time MCRs have rather been considered to be laboratory curiosities, where all starting materials were combined in the same reaction vessel and transformed, without intermediate isolation, into a final product. Many venerable name reactions, such as Hantzsch dihydropyridine synthesis (Hantzsch, 1881) or Gewald amino thiophene synthesis (Huang and Dömling, 2011), have evolved to evergreens in heterocyclic chemistry. But it was not before 1959, when Ivar Ugi introduced his four-component extension of the Passerini synthesis of α-acyloxyamides (Banfi and Riva, 2004) to α-aminoacylamides, i.e., peptoids, and recognized the powerful synthetic concept of multicomponent reactions as an enormous development potential for combinatorial chemistry, diversity-oriented synthesis, and, thereby, for the exploration of structural and functional space (Ugi, 1997; Dömling and Ugi, 2000; Ugi et al., 2003). As a consequence MCRs have become a valuable tool for the preparation of all kinds of functional molecules.

MCRs are often considered as one-pot methodology, which sets the first prerequisite (Posner, 1986; Tietze and Beifuss, 1993; Tietze, 1996; Hulme and Gore, 2003). This experimental setup warrants high convergence, high diversity, and preferentially easily available starting materials for enabling the explorational potential. In its earliest form, MCRs have been assigned to be domino processes, where all starting materials are introduced from the beginning of the process. However, according to Tietze’s more general definitions (Tietze and Beifuss, 1993; Tietze, 1996), which also encompass Posner’s one-pot approach (Posner, 1986), the quite narrow domino approach can be considerably expanded by also allowing stepwise addition of substrates and further additives (catalysts, cosolvents, and effectors) by maintaining the initial conditions (temperature and pressure) in a sequential fashion or by altering them in a consecutive approach. All these three scenarios—domino, sequential and consecutive MCRs—fulfill the *in sensu stricto* definitions of all one-pot methodologies: all transformations proceed in the same reaction vessel without a change of the reaction medium, three or more reactants are employed to form two or more new bonds, and a significant number of atoms from the starting materials are embedded in the product. All this makes MCRs *per se* highly atom economical (Trost, 1991; Trost, 1995; Sheldon, 2000). The practical aspect of the one-pot concept avoids intermediate workup as well as purification after each reaction step, finally leaving a single purification process after completion of the sequence.

Besides the enormous practical aspects of MCRs the underlying reactivity principle is the perpetual generation of functional groups and their selective transformation according to their relative reactivities. (Müller, 2014). As clearly outline by Tietze (Tietze and Beifuss, 1993; Tietze, 1996), the exploratory potential for developing new synthetic methodologies based upon one-pot transformations lies in the sophisticated combination of elementary processes and reactivities that can be either polar or unpolar, concerted processes proceeding via pericyclic transition states, radical and photochemical processes, and the vast manifold of organometallic reactivity in stoichiometric or catalytic fashion.

In the Research Topic “*Multicomponent Reactions (MCRs) Towards Scaffolds with Versatile Applications*” we have compiled various aspects of modern MCR chemistry. Three contributions place a special emphasis on methodological developments by metal-free MCR syntheses of trifluoromethyl-1,2,4-triazole scaffolds (Wang et al.), by Pd-catalyzed asymmetric MCR synthesis of α-arylglycine derivatives (Jakob et al.), and by summarizing advancements of metal-mediated MCR syntheses in general (Sakthivel et al.). The fourth contribution takes a conceptual approach and summarizes and outlines the application of MCRs for accessing chromophores, which as functional π-systems are the molecular key constituents in photonic and electronic applications (Brandner and Müller). We hope that this Research Topic will inspire to follow the exciting path of MCRs, which inevitably have become a playground for developing superior, sustainable methodologies allowing to tackle scientific challenges with tailored molecules in a broad scope from life to materials sciences.
